# Spin-torque devices with hard axis initialization as Stochastic Binary Neurons

**DOI:** 10.1038/s41598-018-34996-2

**Published:** 2018-11-12

**Authors:** Vaibhav Ostwal, Punyashloka Debashis, Rafatul Faria, Zhihong Chen, Joerg Appenzeller

**Affiliations:** 1 School of Electrical and Computer Engineering, Purdue University, West Lafayette, IN 47907 USA; 2 Birck Nanotechnology Center, Purdue University, West Lafayette, IN 47907 USA

## Abstract

Employing the probabilistic nature of unstable nano-magnet switching has recently emerged as a path towards unconventional computational systems such as neuromorphic or Bayesian networks. In this letter, we demonstrate proof-of-concept stochastic binary operation using hard axis initialization of nano-magnets and control of their output state probability (activation function) by means of input currents. Our method provides a natural path towards addition of weighted inputs from various sources, mimicking the integration function of neurons. In our experiment, spin orbit torque (SOT) is employed to “drive” nano-magnets with perpendicular magnetic anisotropy (PMA) -to their metastable state, i.e. in-plane hard axis. Next, the probability of relaxing into one magnetization state (+m_i_) or the other (−m_i_) is controlled using an Oersted field generated by an electrically isolated current loop, which acts as a “charge” input to the device. The final state of the magnet is read out by the anomalous Hall effect (AHE), demonstrating that the magnetization can be probabilistically manipulated and output through charge currents, closing the loop from charge-to-spin and spin-to-charge conversion. Based on these building blocks, a two-node directed network is successfully demonstrated where the status of the second node is determined by the probabilistic output of the previous node and a weighted connection between them. We have also studied the effects of various magnetic properties, such as magnet size and anisotropic field on the stochastic operation of individual devices through Monte Carlo simulations of Landau Lifshitz Gilbert (LLG) equation. The three-terminal stochastic devices demonstrated here are a critical step towards building energy efficient spin based neural networks and show the potential for a new application space.

## Introduction

Emerging spintronic devices have recently attracted attention for efficient implementation of more-than-Boolean computational systems such as neural networks^1^, Bayesian networks^2–4^, Ising networks^4–6^, and invertible logic^7^. Key to the implementation of such systems is the stochastic nature of the network building blocks^8–10^ - nano-magnets in this demonstration – in response to an external stimulation. The desired output characteristics display a sigmoidal probability to find the nano-magnet in one or the other magnetization state – here as a function of an input current. One approach is to drive the nano-magnet into its metastable state through hard axis initialization and let it relax in the presence of an input current, which determines the probability of the nano-magnet settling to one of the states, (+m_i_) or (−m_i_)^4,11^. Here, we will present proof-of-concept spin-devices that employ SOT for hard-axis initialization and a current induced Oersted field to control their output states. Spin devices using this approach emulate the functionality of a stochastic binary neuron with the average output modelled by equation $y=f(\sum _{j}{w}_{ij}{x}_{j}+{b}_{i})$, where *f* is the sigmoidal function, w_ij_ is the synaptic weight corresponding to the input node x_j_, and b_i_ is the default bias. Along this idea, directed networks consisting of spin devices with weighted interconnection can be demonstrated.

In heavy metal (HM)/ferromagnet (FM) systems, spin orbit torque (SOT) switching through the spin Hall effect (SHE) in the HM is an efficient method to control the magnetization of the FM^12,13^. However, since the generated spin is always polarized along the surface plane, magnets with perpendicular magnetic anisotropy (PMA) cannot be deterministically switched. A symmetry breaking in-plane magnetic field is therefore required for SOT switching in HM/ PMA-FM stacks^12^. However, if deterministic switching is not required, currents at levels beyond those typically required for field-assisted switching can drive PMA magnets into their metastable in-plane magnetization states through SOT^14,15^. Once the SOT current is removed, the PMA magnet can relax back to one of the two stable states, (+m_i_) and (−m_i_), with a 50%/50% probability in a purely random fashion that is entirely determined by thermal noise. In this way, a true “random number generator” is created. On the other hand, if the SOT current is removed while a small external perpendicular magnetic field is present, a magnetization in the same direction as the applied field will be preferred with a probability distribution determined by the field strength. To generate a local, perpendicular Oersted field, a small metal loop that is isolated through a SiO_2_ layer from the nano-magnet is used in our device layout as the input terminal. In this way, input-output isolation is ensured, which is one of the requirements to interconnect building blocks into networks for the applications proposed in^14^. In order to show that a spin device is a natural analogue to a stochastic binary neuron, two of these devices are interconnected in this article to form a directed network, and their basic operations are demonstrated.

## Experimental Results

**Figure 1:**
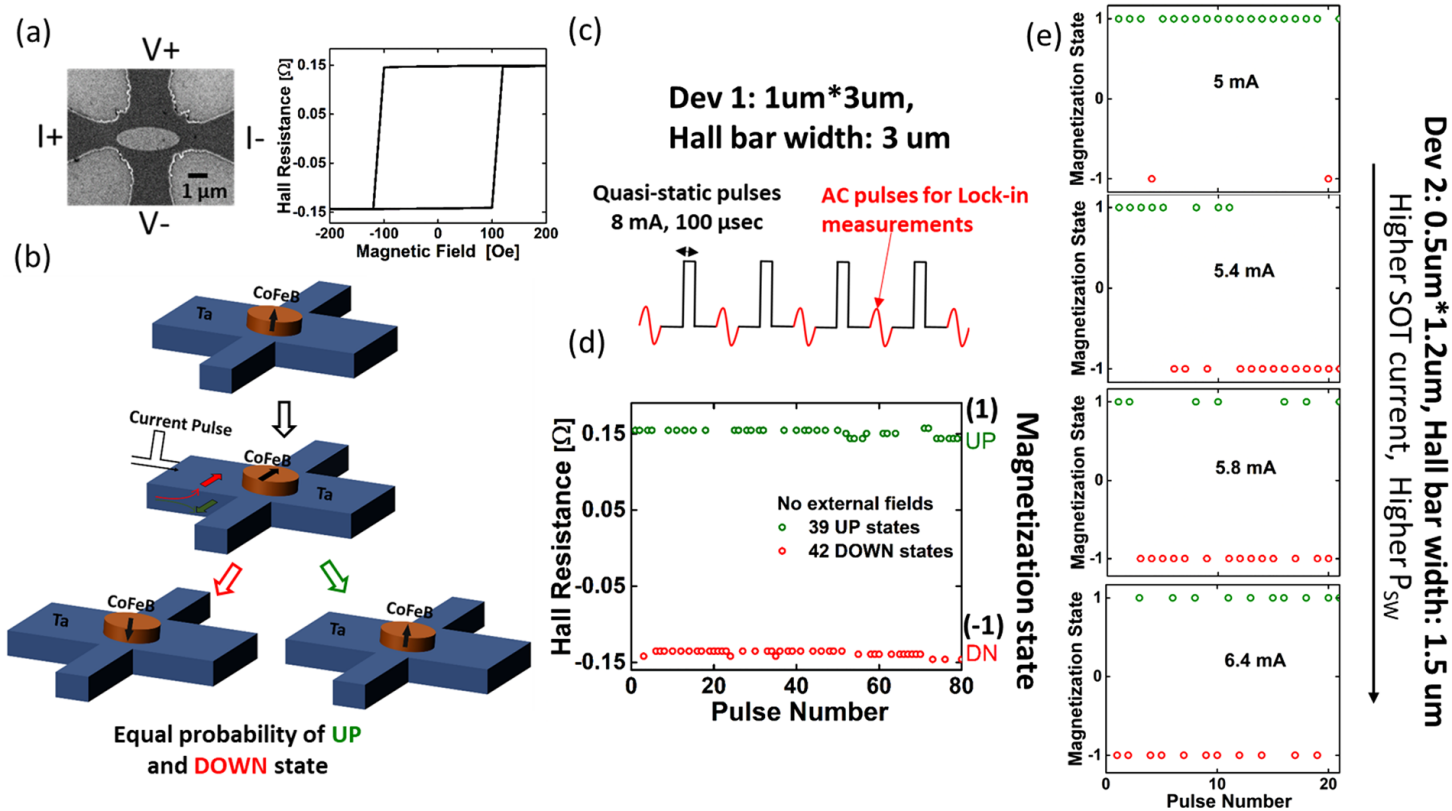
(**a**) SEM image of a readily fabricated device and B-field dependent AHE loop. (**b**) Device operation with SOT hard axis initialization. (**c**) Experimental measurement scheme consisting of a quasi-static current pulse through the Hall bar for hard axis initialization, followed by an AC current to measure the magnetization state. (**d**) Magnetization states of device 1, after each SOT pulse showing its stochastic nature. (**e**) Magnetization states of device 2, after each SOT pulse for different current amplitudes.

A material stack of Ta(7)/CoFeB(1)/MgO(2)/Ta(2) (numbers in brackets denote the respective film thicknesses in nm) was defined on a Si/SiO_2_ substrate using a physical vapor deposition sputter system at a base pressure of 3*10^−8^ Torr. The CoFeB thickness of 1 nm was chosen to induce PMA and confirmed by measuring the magnetic moment (M) versus magnetic field (H) loop of an un-patterned film using a SQUID measurement set-up. The film stack was patterned into Hall bars by e-beam lithography using a bilayer resist stack of polymethyl methacrylate (PMMA) and hydrogen silsesquioxane (HSQ), followed by Argon (Ar) ion beam etching down to the SiO_2_ surface. Next, elliptical PMA magnets of various sizes ranging from 0.5 um*1.2 um to 1 um*3 um were patterned using the same lithography and etching process as before until the bottom Ta (7 nm) layer was reached. Last, electrical contacts were defined using e-beam lithography and a lift-off process after depositing Ti (20 nm)/Au (100 nm) metal stacks. Figure 1(a) shows a scanning electron microscopy (SEM) image of a fabricated device as well as a schematic illustration of the device during the measurement procedure described in the previous paragraph. An elliptical shape was chosen so that the nano-magnet covered the width of the voltage arm entirely and at the same time was located far enough from the edges of the current arm of the Hall bar to minimize the out-of-plane Oersted field due to currents through the Hall bar.

To detect the magnetization state of the PMA nano-magnet, the anomalous Hall effect (AHE) is used. Figure 1(a) shows an exemplary AHE measurement versus out-of-plane magnetic field, clearly showing the expected hysteresis loop and the change in the polarity of the AHE signal when the PMA magnetization switches from up to down and vice versa.

Note that an offset in the Hall resistance vs. magnetic field (H) loop has been removed in this and all subsequent plots. The AHE resistance was measured using a lock-in set-up with an AC current of 100 uA at a frequency of 97.7 Hz. SOT driven deterministic switching in the presence of an in-plane field was performed using quasi-static 100 us long dc pulses. Figure S1(a) in the Supplementary Information (SI) shows AHE vs. DC current with the assistance of an in-plane field of 20 mT. The direction of the loop reverses (changes from clockwise to counter clockwise) when the magnetic field polarity is reversed (see Fig. S2(a) in SI), which is a signature of SOT driven magnetization switching^12,16^.

On the other hand, in the absence of an in-plane magnetic field and with DC current pulses of 7 mA applied on Dev 1, the nano-magnet retained its magnetization states with the AHE signal remaining the same after every pulse (see Fig. S1(b) in SI). This situation changes once the DC current levels reach 8 mA (see Fig. 1(c)). The AHE resistance is found to be positive or negative, corresponding to the magnetization pointing upwards (UP) or downwards (DN), respectively. In fact, performing 81 such measurements, the signal showed 39 times positive and 42 times negative values as shown in Fig. 1(d), consistent with the expectation of a 50% probability to find the PMA after the SOT pulse in its UP (+1) or DN (−1) state. Moreover, only two distinct AHE values (±0.15 Ω) were detected, implying that the PMA magnet responded as a “whole” rather than breaking into multiple magnetic domains during the pulsing procedure. Figure 1(e) shows the magnetization states of another device (Dev 2) with a nano-magnet of size 0.5*1.2 um^2^ after SOT pulses of 5 mA, 5.4 mA, 5.8 mA, and 6.4 mA with no external field applied. Similar to Dev 1, a sufficiently large SOT current is needed to achieve the desired stochasticity. It is interesting to observe that the probability of switching (P_SW_) increases with increasing SOT current amplitude but independent of its polarity, as shown in Fig. S2(b,c). With sufficient statistics, P_SW_ eventually saturates at 50% when the threshold current for the meta-stable initialization is reached^14^. All current levels above this threshold level, generate random binary-states of the PMA magnet^14^. Note that the current required for initializing Dev 2 is smaller than for Dev 1 due to a smaller Hall bar width. While random choices of the two resistance states are demonstrated here, it is understood that in order to apply this scheme to create a true random number generators (RNGs), the “NIST Statistical Test Suite” needs to be satisfied^17^. With the limited data size from our experiments, we are currently unable to perform all NIST tests, but can only employ those that are appropriate for small data sets. Table S3 shows results of 4 NIST tests on the data shown in Fig. 1(d) and provides initial evidence for the high quality of our RNGs. Even though we have used electrical pulses of 100 us, due to the details of our measurement set-up, devices can in principle achieve high operational speed and are expected according to ref.^14^ to be able to operate with electric pulses of a few nanoseconds. Also note that other approaches of implementing RNGs, e.g. by means of Spin Transfer Torque (STT) Magnetic Tunneling Junctions (MTJs)^18^, require “fine tuning” of individual devices to achieve a 50% P_SW_ and thus are expected to show significant device-to-device variations. Moreover, MTJ-based devices typically require a “RESET” current pulse for re-initialization. On the other hand, our approach is robust in the sense that even though the threshold current for hard-axis initialization may vary from device-to-device, one can always choose a sufficiently large current to initialize all devices^14^.

**Figure 2:**
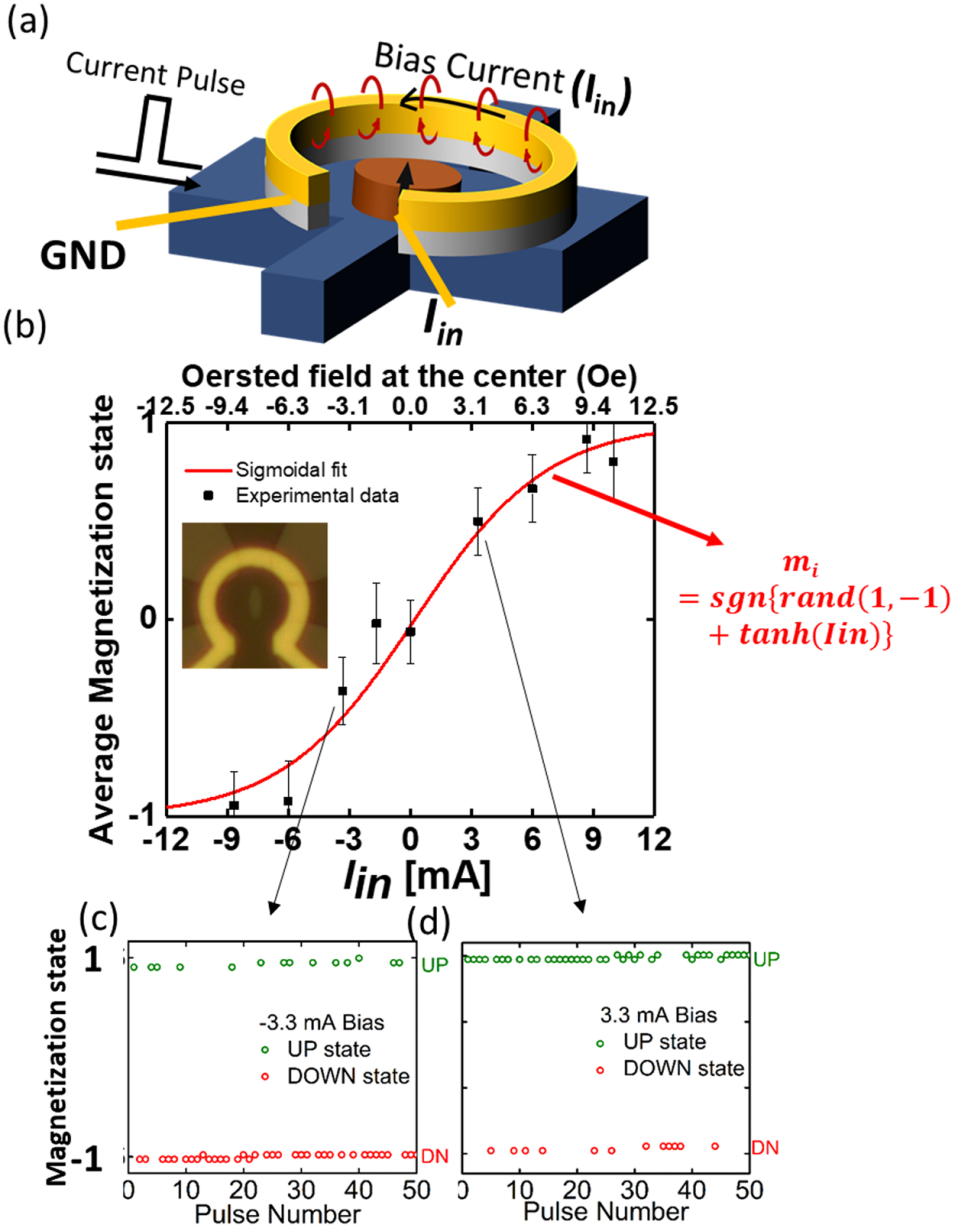
(**a**) Cartoon of an all-electrical device with Oersted field generating metal ring to control the final magnetization state after an SOT pulse. (**b**) Average magnetization state of the device (Dev 1) for different currents (I_in_) through the Oersted ring. (**c**,**d**) Magnetization states after each SOT pulse for I_in_ = −3.3 mA & +3.3 mA.

Next, the tunability aspect of our RNGs will be discussed. In order to impact the final probability of finding a device in its UP or DN state, a ring shaped Ti (10)/Au (80) metal loop was designed around the nano-magnet as shown in Fig. 2(a). This metal loop was isolated from the underlying Hall bar by a 120 nm thick SiO_2_ layer. Passing current through the loop was employed to generate a symmetry breaking Oersted field, which allows tuning the probability of the final magnetization state after releasing the SOT current. The out-of-plane Oersted field generated at the location of the nano-magnet is approximately given by ${\mu }_{0}I/2r\,$*(eq.1)* and was kept “on” during the SOT pulses. ~12 Oe field could be generated by passing 10 mA current through the loop, which was sufficient to entirely pin the magnetization in the UP or DN state with a ~100% probability. Figure 2(b) shows the average magnetization (averaging over either 72 or 51 pulses) as a function of the current applied to the loop (*I*_*in*_), displaying the expected sigmoidal shaped curve taking into account more than 600 measured data. Figure 2(c,d) show exemplary magnetization states measured at *I*_*in*_ of −3.3 mA and +3.3 mA, which resulted in the respective data points in the main plot. The expected binomial standard deviation $\sigma$ is calculated using the equation: $\sigma =\sqrt{\frac{p(1-p)}{n}}$ where *p* is the measured probability of magnetization state being UP (+1) and *n* is the number of pulses (sample size) used to estimate p. Both, the average magnetization (2*p-1) as well as the error bars calculated according to $\sigma \ast 2$ are displayed in Fig. 2(b). Since the expression for $\sigma$ is not valid when p approaches 0 or 1, error bars are truncated when the average magnetization state is close to −1 and +1. To our knowledge, this is the first demonstration of a current controlled spin device with tunable stochasticity and input-output isolation!

**Figure 3:**
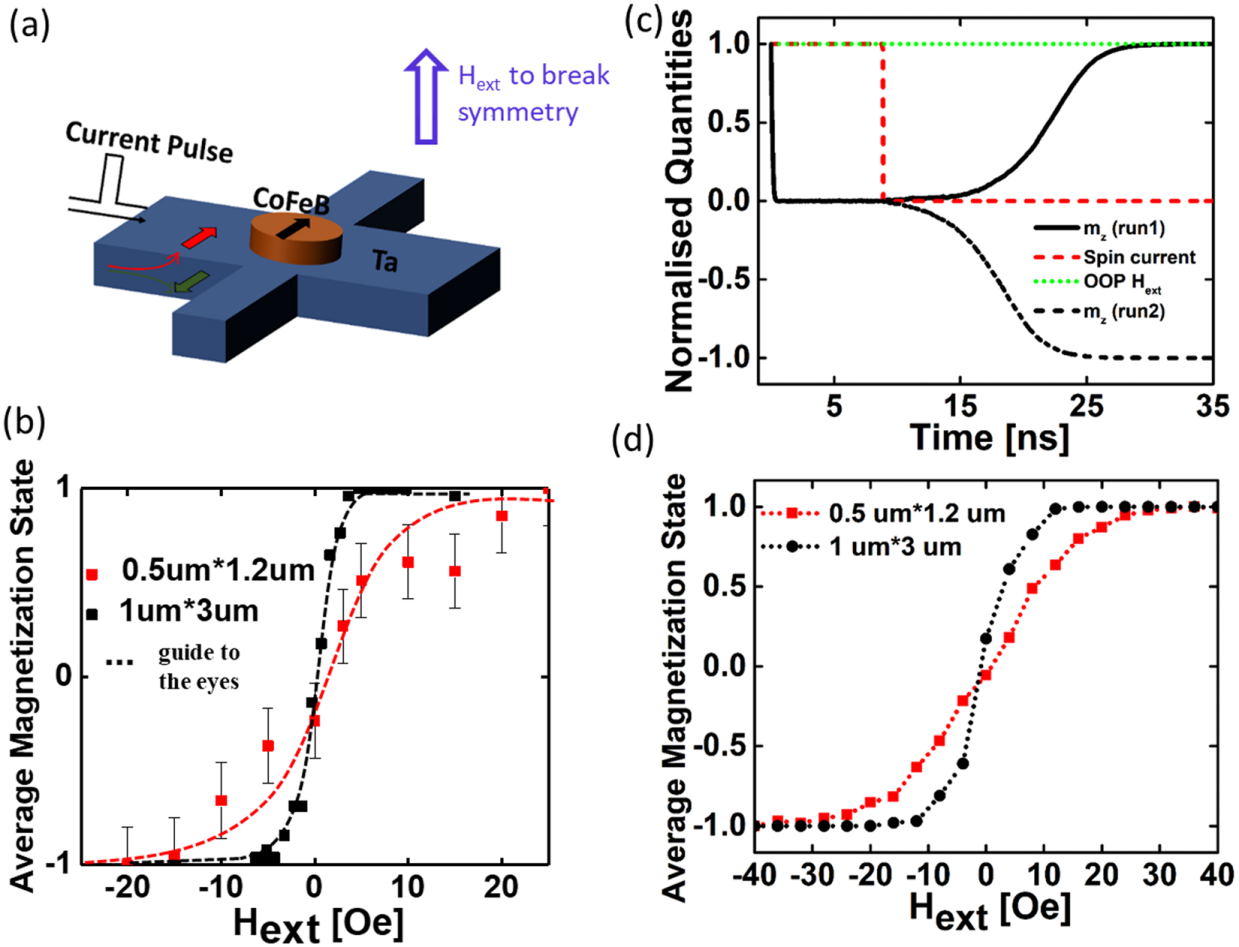
(**a**) Experimental set-up to measure average magnetization under external magnetic field (H^ext^). (**b**) Average magnetization state of the device under H_ext_ - impact for two different magnet sizes. (**c**) sLLG simulations showing the magnetization dynamics. (**d**) sLLG simulation results for magnets of sizes as shown in (**b**).

To complement the above demontration, we also performed measurements where the tunability of the RNG was accomplished by means of an external out-of-plane magnetic field rather than the locally generated Oersted field. A schematic illustrating the measurement is shown in Fig. 3(a). As expected, similar sigmoidal curves of the average magnetization as a function of external magnetic field were obtained for two different magnet sizes. Each data point in Fig. 3(b) is a result of 41 independent measurements. For the nano-magnet with dimensions of 1 um*3 um, saturation of the average magnetization occurred at a magnetic field of ~+5/−5 Oe, while a field of +20/−20 Oe was required for the smaller magnet with dimensions of 0.5 um*1.2 um (Note that in some instances the device characteristics did not show a perfect symmetry relative to zero magnetic field presumably due to a slight misalignment of the nano-magnet relative to the tantalum Hall bar which results in an out-of-plane Oersted). While a perfect quantitative match is not expected, since the magnetic field lines at the nano-magnet location are not identical for the experiment with the Oersted ring and the global external magnetic field, the results in Figs 2(b) and 3(b) are in reasonable agreement.

To evaluate these findings, time-resolved simulations of mono-domain nano-magnets using stochastic Landau-Lifshitz-Gilbert (sLLG) equation have been performed. Even though for the magnet sizes used in the experiments, the sLLG assumptions, e.g. mono-domain behavior and abrupt “input current turn-off” do not strictly apply, the simulations provide valuable insights into how the sigmoid curve gets effected by magnetic properties such as volume (V), magnetization moment (M_s_), and perpendicular magnetic anisotropic field (H_k_). Note that for a qualitative description the assumption of mono-domain switching behavior has been previously successfully applied to describe SOT switching^19^. As in the experiment, a spin-current pulse is applied for a certain time (dashed red line in Fig. 3(c)) to drive the magnet into its in-plane hard axis, while a small symmetry breaking out-of-plane magnetic field is simultaneously applied (green dashed line in Fig. 3(c)). Figure 3(c) shows the dynamic response of the nano-magnet’s z-component of the magnetization, m_z_, before and after the spin-current pulse has been removed. As apparent from the black solid and dashed line, different simulation runs do indeed show that for the same conditions – in terms of symmetry breaking magnetic field – the magnet can settle in the UP or DN state after initially having its magnetization in-plane. However, as shown in Fig. 3(d) the probability depends critically on the exact H_ext_-value. Parameters used in the simulation are M_s_ = 1000 emu/cc, H_k_ = 200 mT, nano-magnet thickness (t) = 1 nm and lateral sizes as shown in the figure legend. Each data point in Fig. 3(d) represents an average of 200 sLLG simulation runs. Consistent with the experimental findings, smaller nano-magnets require a higher magnetic field to reach the condition that the magnet pins with a 100% probability to one of its two states (UP or DN). After initializing the nano-magnet to its metastable state, the probability to settle in either the +1 or −1 states will be dependent on the energy difference between the +1 or −1 state due to the external field. For the same external field, magnets with higher M_s_*V product will result in a larger energy difference and hence, larger magnets will exhibit a sharper probability curve with respect to an external magnetic field. Figure S4 in the Supplementary Information section shows sLLG simulation results for nano-magnets of the same size but with different H_k_. Higher H_k_-values results in a “sloppier” probability curve. Controlling the slope of the probability sigmoidal curve becomes important, in order to properly tune the device characteristics to be most desirable for a particular stochastic spin device application. For example, to generate true random numbers with equal probability of 0 and 1, smaller size nano-magnets that exhibit a high H_k_ will be useful, since those display a “sloppier” probability curve and will be less susceptible to stray fields. On the other hand, larger nano-magnets with low H_K_ will be preferred for applications that require modulation of the probability from +1 to −1 with minimal change of input.

**Figure 4:**
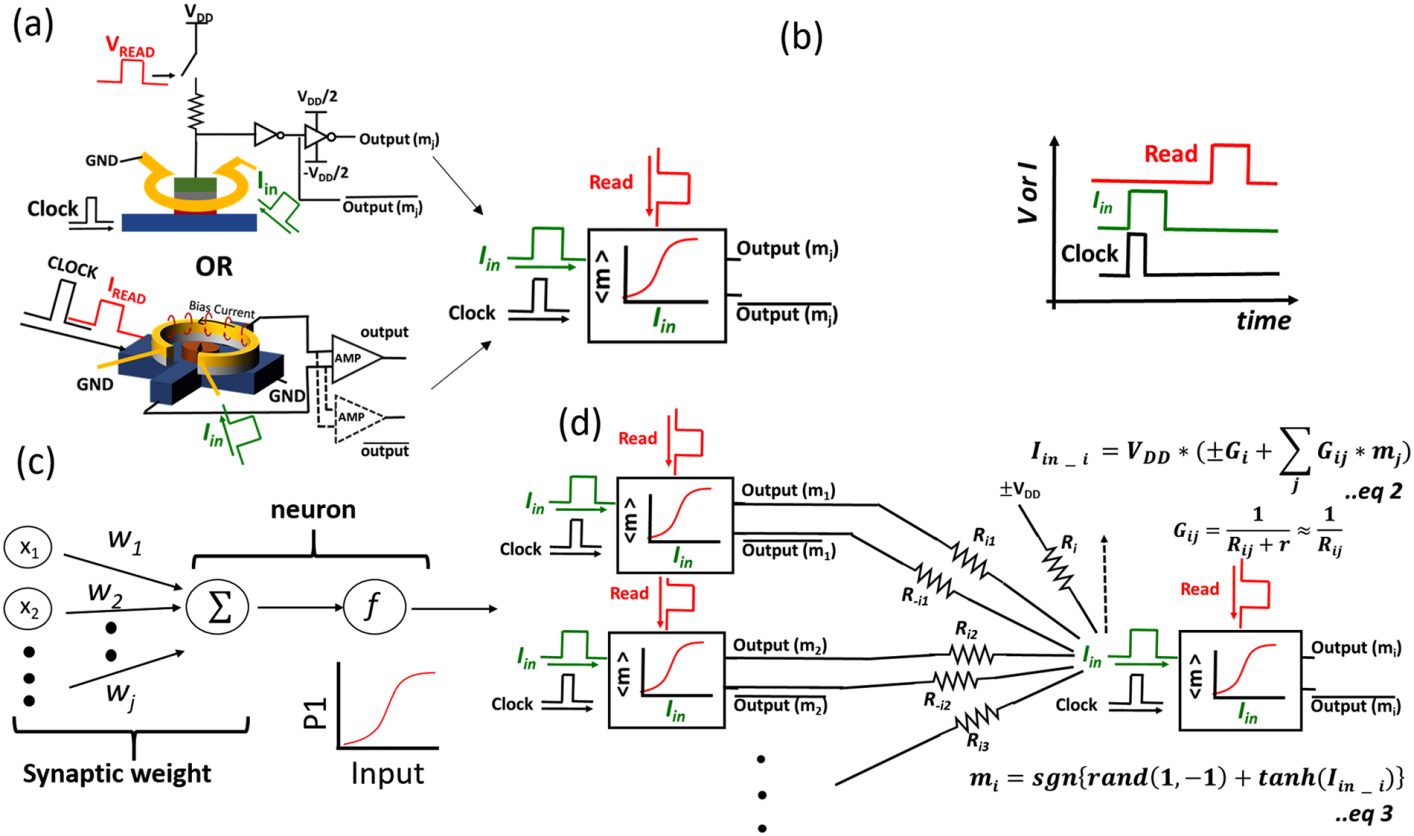
(**a**) Two stochastic spin devices as described in the text used as binary stochastic neurons. (**b**) Electrical inputs to the stochastic device, i.e. clock, I_in_ and READ. (**c**) Nodal representation of the neural network. (**d**) Neural network implementation with spin devices as stochastic binary neurons and resistive weight network.

Last, the stochastic spin devices demonstrated above can be employed as stochastic binary neurons to be interconnected to form a neural network – similar to the ones proposed in ref.^20–22^, with its nodal representation shown in Fig. 4(c). In such a network, each node (one spin device) will exhibit a sigmoidal activation function with inputs (in our case provided through the Oersted ring) from the nodes of previous layers as shown in Fig. 4(d). Layers will be updated sequentially. Preferably, the magnetization state of the given node will be read using a magnetic tunnel junction (MTJ) in series with two inverters as shown in the top of Fig. 4(a). While the READ operation can also be performed using AHE, as we will demonstrate in the following experiment, it does require voltage amplification due to the relatively low achievable voltage amplitude. Two inverters in series (see Fig. 4(a) top) provide *output* and $\overline{output}$, which are both required to realize positive and negative weights in the network. Electrical inputs, i.e. clock, I_in_ and READ of the given node were applied according to Fig. 4(b). The clock (in our case an SOT pulse) is used to drive nodes (nano-magnets) into their metastable states in the given layer. During the SOT pulse, outputs from previous layers weighted by a resistance network (synaptic weights) are provided to the metal loops of the nodes in the current layer as an input. The input current from node “j” of the previous layer to node “i” of the next layer, connected through the resistance $\,{R}_{ij}$ is defined by ${I}_{in-ij}=\frac{{m}_{j}\,\ast \,{V}_{dd}}{{R}_{ij}+r}$, where $r$ is the resistance of Oersted ring and ${m}_{j}$ is the magnetization state of node j (+1 or −1). For the case of a low resistance Oersted ring $(r)$ as in our experiment, this input current can be approximated as $\,{I}_{in-ij}\approx \frac{{m}_{j}\,\ast \,{V}_{dd}}{{R}_{ij}}$. Input currents from multiple nodes “j” will be added at the Oersted ring of node “i” providing a total input current of ${I}_{in-i}=\sum _{j}\frac{{V}_{dd}\,\ast \,{m}_{j}}{{R}_{ij}}$. If comparing this expression with the one for a stochastic binary neuron - $(\sum _{j}{w}_{ij}{x}_{j}+{b}_{i})$, where w_jj_ is the synaptic weight corresponding to the input node x_j_ and b_i_ is the default bias, one can identify the synaptic weight between two neurons for the network shown in Fig. 4(d) as $\frac{{V}_{dd}}{{R}_{ij}}={V}_{dd}\ast {G}_{ij}$. Since both *output* and $\overline{output}$ are available for the given nodes, negative weights can be realized by using the weight connection corresponding to $\overline{output}$. Equation 2 in Fig. 4(d) describes the weighting and summation operation of inputs using the resistor network shown. Equation 3 in Fig. 4(d) displays the sigmoidal activation function of the stochastic binary neuron using this weighted input. In principle one can envision more power efficient implementations of the above neurons by employing other spintronic phenomena, such as voltage controlled magnetic anisotropy (VCMA) and Spin Transfer Torque (STT). Compared to SOT, VCMA would potentially require a much lower energy for hard axis initialization of the nano-magnets as proposed in^20^, while after the initialization, STT (instead of an Oersted field) could be used to efficiently tune the probability of the final magnetization state^21^.

**Figure 5:**
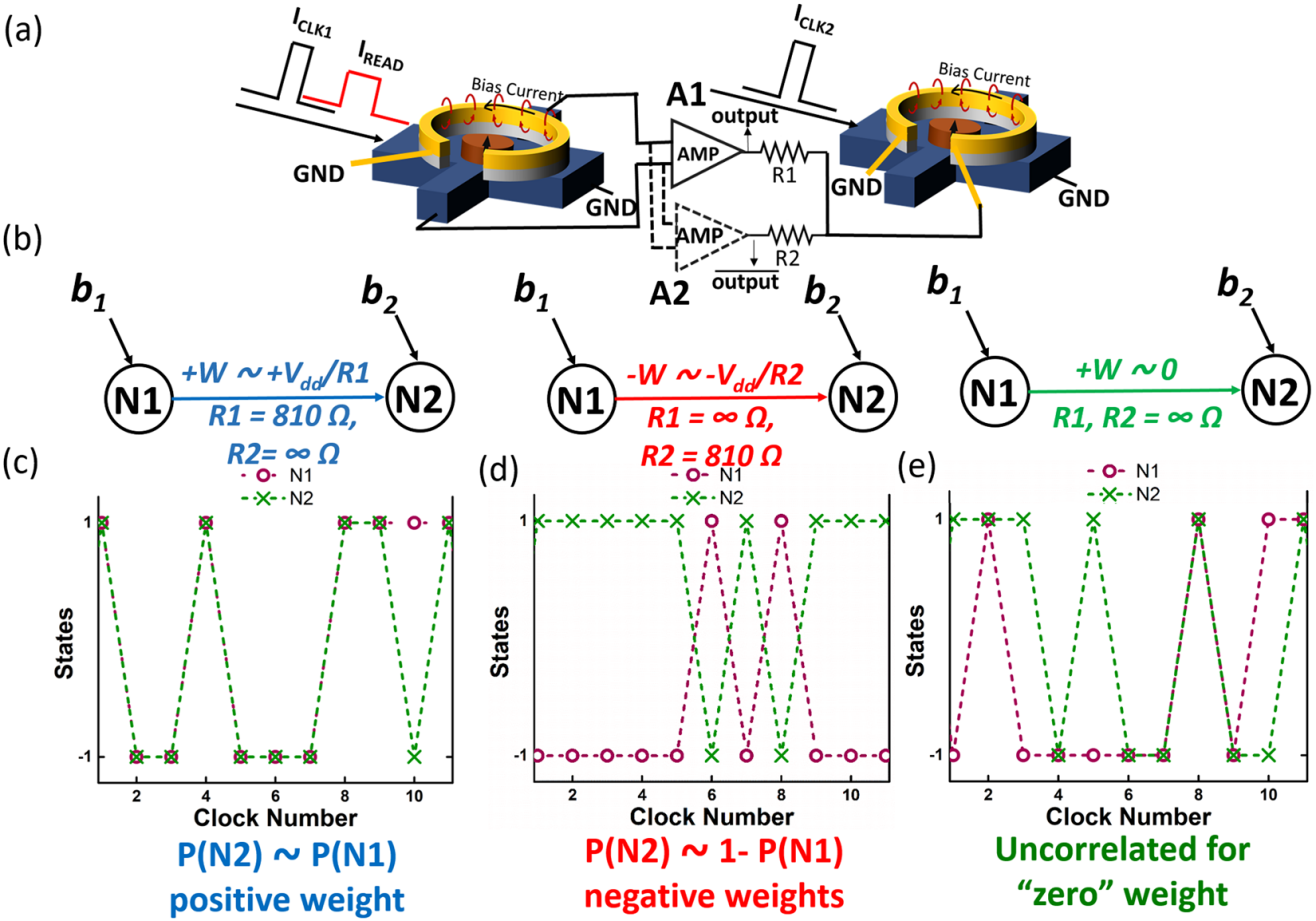
(**a**) Experimental set up for two interacting devices (**b**) nodal representation of + and − connections (**c**–**e**) experimental measurements for +, − and 0 weights.

Based on the above neuromorphic system, we have demonstrated a two-node network to show the control of an output node by an input node and a weighted interconnection between them. The experimental set-up of the measurements performed is shown in Fig. 5(a) where two stochastic spin devices were used. The input clock 1 was used for hard axis initialization of device N1. After the SOT pulse was removed (after the clocking 1 cycle ended), a small I_READ_ dc current was used to read the magnetization state of N1 using AHE. The AHE voltage corresponding to $\pm m\,\,$is amplified to $\pm {V}_{dd}$ or $\mp {V}_{dd}$ using an amplifier A1 or A2, respectively. Next, the amplifier output voltage was applied to the input “Oersted ring” of the second node N2 through a resistor R1 or R2 as shown in Fig. 4. While I_READ_ was on for node N1, the input clock 2 was applied to node N2 for the hard axis initialization, i.e. an SOT pulse was applied to node 2. Hence, node N2 received an input from the previous node N1 while being in its metastable state. Using V_dd_ = 10 V and R1 = 810 Ohm and R2 = ∞ corresponds to a current of ~10 mA in the Oersted ring. This input bias current resulted in a “strong” positive connection between the two neurons – see sigmoidal curve in Fig. 2(b). Note that any resistance values of around 1000 Ohm will result in a strong connection. In other words, if the input from the previous node is “+1”, the connection is strong enough to ensure that node 2 will be found in an average magnetization state of “+1” with a probability close to 100%. A negative weight connection is achieved by using R2 = 810 Ohm and R1 = ∞. As explained in the previous paragraph, the strength of the connection can be modified by changing the resistance used for the connection. This is obvious from the case of an infinite resistor R1 and R2 – which means zero weight connection between N1 and N2. Figure 5(b) is the nodal representation of the network for positive weight, negative weight and zero weight connections. As a result, the output of the node N2 is now a function of the input from the previous node N1 and the weight of the connection. For a positive connection, node N2 follows the magnetization state of node N1 as shown Fig. 5(c) (COPY operation). For clock number 10, N2 doesn’t follow the magnetization of N1 which means that the connection strength is not strong enough to ensure a “perfect” correlation. For a negative weight connection, node N2 follows the opposite magnetization state of node N1 Fig. 5(d), while for zero weight connection, the outputs of N1 and N2 become uncorrelated as shown in Fig. 5(e). In other words, the experimental data in Fig. 5(c) unambiguously show that the probability (P_N2_) to find node N2 in a certain magnetization state is dependent on the probability (P_N1_) of node N1 and the weight of the connection. P_N2_ is approximately either P_N1_ or 1 - P_N1_, depending on whether a strong positive or negative weighted connection is used.

## Conclusion

As CMOS scaling is reaching its limits, more-than-Boolean computational systems using novel device concepts are being explored for computational advancement. Here we have experimentally demonstrated a new type of stochastic spin devices, which may become the building block for probabilistic computing. The unique properties of our devices, such as a current controlled probabilistic binary output state and input-output isolation can be exploited to perform a weighted summation of multiple input nodes like in stochastic binary neurons. Using these stochastic devices, we have demonstrated a two-node directed network in which the probability of the output node is a function of the input node and the weighted connection between them. This initial experimental demonstration shows the potential of stochastic spin devices as building blocks for directed networks with probabilistic outcomes such as Neural networks and Bayesian networks.

## Electronic supplementary material

Supplementary Information
