# Supplementary material for: Spin-torque devices with hard axis initialization as Stochastic Binary Neurons
Source: Sci Rep. 2018 Nov 12;8:16689. doi: 10.1038/s41598-018-34996-2 (PMC6232168; doi:10.1038/s41598-018-34996-2)

Spin-torque devices with hard axis initialization as Stochastic Binary Neurons

Vaibhav Ostwal, Punyashloka Debashis, Rafatul Faria, Zhihong Chen, Joerg Appenzeller

SUPPLEMENTARY INFORMATION

Fig S1: (a) In-plane magnetic field assisted SOT switching of Dev 1. (b) If no external magnetic field is applied, the nano-magnet (Dev 1) retains its magnetization for current pulses of 7 mA. However, for current pulses of 8 mA, the nano-magnet starts to settle either in its +1 or -1 magnetization state.

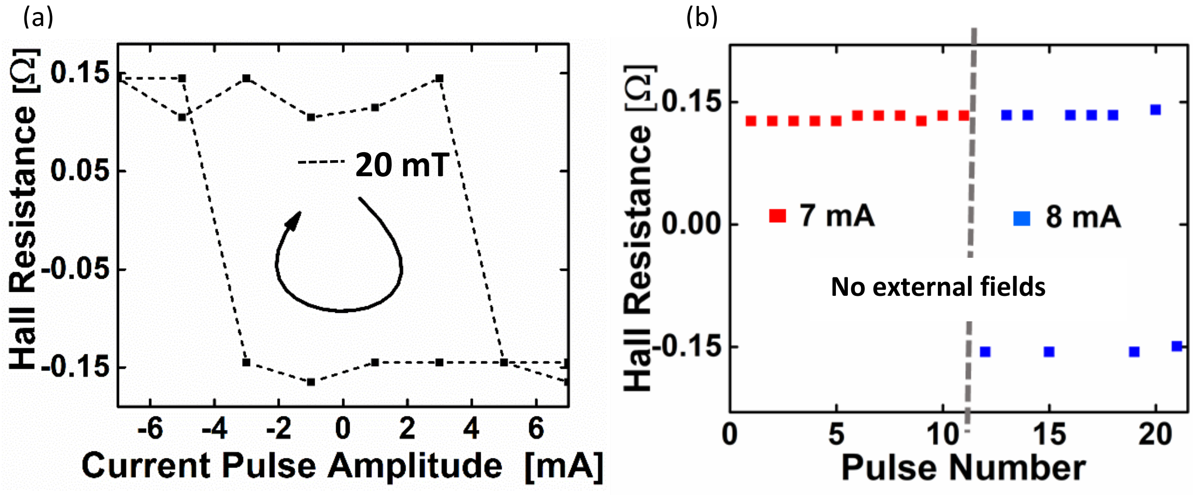

Fig. S2: (a) In-plane magnetic field assisted SOT switching of Dev 2 showing reversal of switching loop for positive and negative magnetic fields (b) Nano-magnet (Dev 2) shows stochastic behavior also for -6.2 mA current pulses (The

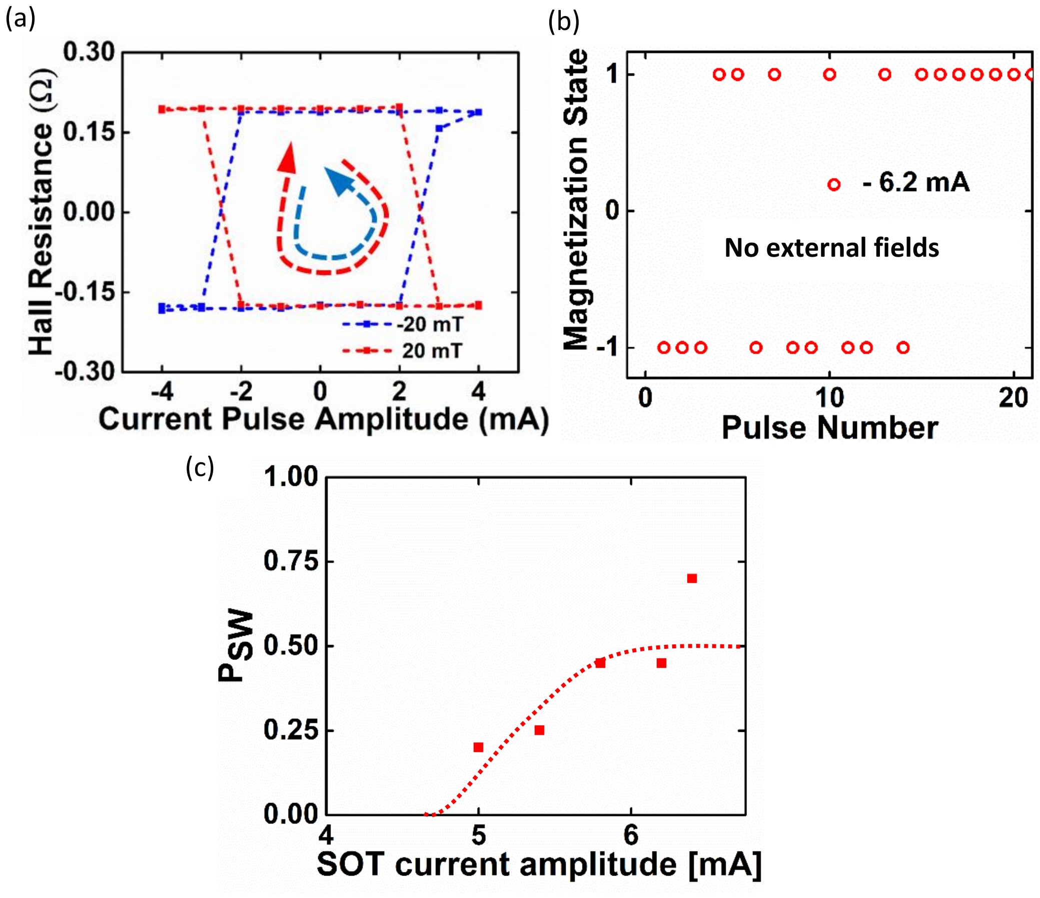

stochastic behavior for positive current pulses is shown in the main text - figure 1(e)). (c) Probability of switching if no external magnetic field is applied: P_{SW} should saturate at 0.5 when the current pulse amplitude reaches the threshold current for hard-axis initialization. The value of 0.75 observed here is a result of small number of sampling performed in our experiment.

Table: S3: Results of 4 of the NIST tests for the validation of a Random Number Generators (RNG) for the data shown in figure 1(d) in the main text. A p-value larger than 0.01 means that the respective data passed the test.

Test Name	p-value
The Frequency (Monobit) Test	.58
Frequency Test within a Block	.42
The Runs Test	.76
The Discrete Fourier Transform (Spectral) Test	.12

Fig. S4: sLLG simulation results for a nano-magnet with a size smaller than discussed in the text showing saturation of the average magnetization at lower external fields for smaller H_k-values.

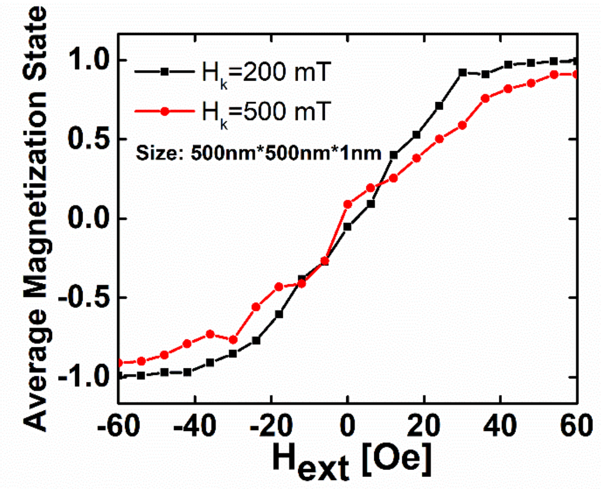

Supplement: Supplementary file 1 — Supplementary Information [file 41598_2018_34996_MOESM1_ESM.pdf]
